# Arrest of Erysipelas

**Published:** 1890-03-08

**Authors:** 


### ARREST OF ERYSIPELAS.

The Kraske-Riedel method of limiting the progress of erysipelas by scarification, with carbolic or sublimate dressings, has scarcely attracted the attention it deserves, though it has been adopted by a number of practitioners on both sides of the Atlantic. Dr. Lauenstein, a distinguished surgeon at Hamburg, advocates it strongly, but with one important modification, viz., that the line or belt of obliquely intersecting (lattice like) scarifications is carried not along the margin of the inflammation, but at a little distance beyond it, where the skin is quite healthy. Under the older plan Dr. Lauenstein is convinced that the frequent failures were owing to the knife acting as an inoculating needle, transferring infection from the diseased to the healthy tissue, and defeating the whole aim of the surgeon.

